# A Rare Case of Posterior Fossa Syndrome Associated with Neuropathic Pain Successfully Treated with a Combination of Gabapentin, Diazepam and Baclofen—A Case Report and Literature Review

**DOI:** 10.3390/children11121410

**Published:** 2024-11-21

**Authors:** Mariateresa Giglio, Alberto Corriero, Teresa Perillo, Giustino Varrassi, Filomena Puntillo

**Affiliations:** 1 Department of Interdisciplinary Medicine, University of Bari and Aldo Moro, 70124 Bari, Italy; alberto.corriero@gmail.com (A.C.); filomena.puntillo@uniba.it (F.P.); 2 Pediatric Unit, Policlinico Hospital, 70124 Bari, Italy; terryperillo@hotmai.com; 3 Paolo Procacci Foundation, 00193 Rome, Italy; giuvarr@gmail.com

**Keywords:** posterior fossa syndrome, medulloblastoma, neuropathic pain, gabapentin, cerebellar mutism

## Abstract

Background: Posterior fossa syndrome (PFS), also known as cerebellar mutism syndrome, occurs in about 25% of pediatric patients undergoing resection of a posterior cranial fossa medulloblastoma. It is characterized primarily by mutism or reduced/impaired speech and may include variable symptoms such as motor dysfunction (apraxia, ataxia, hypotonia), supranuclear cranial nerve palsies, neurocognitive changes, and emotional lability. Long-term multidisciplinary rehabilitation is typically required, with recovery taking approximately six months, though many children experience long-term residual deficits. Neuropathic pain associated with PFS is rarely reported in pediatric patients, and evidence for its management is limited. Methods: This case report describes a 10-year-old boy who developed PFS following incomplete resection of a medulloblastoma. Clinical presentation included mutism, irritability, emotional lability, sleep disturbances, and neuropathic pain localized at the C5 level. The patient was treated with a combination of gabapentin, diazepam, and baclofen. Results: The combined pharmacological approach resulted in successful management of the patient’s neuropathic pain and other symptoms associated with PFS, improving his overall condition. Conclusions: This case highlights the potential effectiveness of a multimodal pharmacological regimen for treating neuropathic pain and associated symptoms in pediatric patients with PFS. Further research is needed to explore optimal treatment strategies for this rare but challenging complication.

## 1. Introduction

Medulloblastomas are one of the most common malignant brain tumors in children, accounting for approximately 20% of all pediatric brain tumors. These tumors primarily affect young children, with a median age of onset around seven years, and show a clear male predominance, with an incidence rate of about 2 to 5 cases per million children annually [1]. The World Health Organization (WHO) classifies medulloblastomas into four molecular subgroups based on genetic characteristics: wingless (WNT-)-activated, sonic hedgehog (SHH-)-activated and TP53 wildtype, SHH-activated and TP53 mutant, and non-WNT/non-SHH (formerly groups 3 and 4) [2]. These subgroups represent distinct biological entities with unique genetic, histological, and clinical profiles that influence prognosis and treatment. For instance, WNT-activated medulloblastomas are associated with more favorable outcomes, while SHH-activated TP53 mutant tumors are more aggressive and often require intensified treatment approaches [3].

Although most medulloblastomas arise sporadically, certain cases are linked to genetic predisposition syndromes, particularly within the SHH-activated group. Notable syndromic associations include Gorlin syndrome (related to PTCH1 mutations) and Li–Fraumeni syndrome (associated with TP53 mutations), underscoring the importance of genetic counseling and surveillance in pediatric neuro-oncology [3]. Advances in molecular classification have paved the way for potential subgroup-specific therapies, targeting the unique biology of each subtype. While most of these therapies are still experimental, they hold promise for more targeted, less-toxic interventions in the future [3].

Medulloblastomas typically originate in the posterior fossa, often arising from the roof of the fourth ventricle or the inferior medullary velum [4]. Due to their anatomical location near essential brain structures such as the cerebellum and brainstem, these tumors frequently cause symptoms related to increased intracranial pressure (ICP) from cerebrospinal fluid (CSF) obstruction, including headache, vomiting, and gait ataxia. The latter is frequently associated with midline cerebellar vermis involvement, impacting motor coordination [5,6].

At diagnosis, approximately 20–25% of medulloblastomas exhibit dissemination within the central nervous system (CNS), which complicates management. While metastatic spread is often asymptomatic, it is typically detected through imaging and CSF analysis [4]. Treatment requires a multidisciplinary approach considering the child’s age, molecular subtype, and risk category [4]. Maximal safe resection followed by adjuvant radiotherapy and chemotherapy is standard, as achieving maximal resection is correlated with better survival [7]. However, complete resection can be challenging due to the tumor’s proximity to critical brainstem and cerebellar structures, particularly in young children for whom preserving neurological function is a priority [8].

One of the most challenging complications following medulloblastoma resection is posterior fossa syndrome (PFS), affecting up to 40% of cases. Also known as cerebellar mutism syndrome, PFS encompasses a range of neurological symptoms, including mutism or severely impaired speech, motor deficits (such as apraxia, ataxia, and hypotonia), emotional lability, and cognitive impairments. Behavioral symptoms such as irritability, inconsolability, and withdrawal significantly impact the psychological well-being of the patient and family, highlighting the importance of comprehensive psychosocial support [9].

The pathophysiology of PFS is incompletely understood, though several mechanisms have been proposed. One major theory posits that surgical trauma to the cerebellum or brainstem, particularly the midline vermis [10,11], plays a crucial role. Another hypothesis suggests that damage to the bilateral dentato-thalamo-cortical pathways, which connect the cerebellum to the motor cortex, is central to PFS development. These pathways, traversing the superior cerebellar peduncles, are essential for motor coordination, cognition, and affective functions. The disruption of these pathways may result in mutism, motor deficits, and other characteristic PFS symptoms [12,13]. Additional factors, including postsurgical edema, neurotransmitter imbalances, and focal vasospasm, likely compound the primary injury and further complicate PFS pathogenesis [14,15].

Advances in neuroimaging, particularly diffusion tensor imaging (DTI), have enhanced our understanding of PFS by enabling the visualization of cerebellar–cerebral pathways. DTI studies have shown that the degree of disruption in the dentato-thalamo-cortical tracts correlates with clinical severity, offering a valuable diagnostic tool. Identifying these disruptions pre-operatively may inform surgical planning, as preserving critical pathways could help reduce the risk of PFS [16].

Several risk factors are associated with a heightened likelihood of developing PFS, including younger patient age, surgeries conducted at low-volume centers (indicating possibly lower surgical experience), central tumor location, and specific molecular subtypes [4,17]. Symptoms of PFS generally appear within a few days post surgery, suggesting a delayed cascade of neurophysiological changes triggered by initial trauma. The cerebello-cerebral diaschisis theory describes this process as involving reduced cerebral blood flow, neurotransmitter dysregulation, and axonal injury. Hypertrophic olivary degeneration, another secondary mechanism linked to PFS, reflects the cerebellum’s complex role in regulating motor, cognitive, and emotional functions [18,19]. Therefore, avoiding the aggressive intra-operative manipulation of cerebellar structures has been proposed to reduce the risk of post-operative PFS [20]. Moreover, cerebello-cerebral diaschisis and hypertrophic olivary degeneration cannot be detected by conventional magnetic resonance imaging sequences, instead requiring advanced MRI techniques, such as diffusion tensor imaging or brain network analysis [21,22].

The recovery trajectory for PFS varies widely. Some children begin to regain speech within six months, while others require longer rehabilitation, particularly for motor functions such as gait [23]. Long-term cognitive and neurobehavioral sequelae, including deficits in processing speed, working memory, executive functioning, and attention, are common and can impair academic and social development [9,24]. These lasting effects underscore the importance of early intervention and comprehensive, multidisciplinary rehabilitation. Effective management often involves a team-based approach, combining speech and occupational therapy, mood stabilization medication, and psychosocial support. Despite these efforts, evidence-based treatment guidelines for PFS are limited, and severe cases present ongoing challenges for clinicians and families [25].

An underreported yet significant complication of PFS is neuropathic pain, which complicates both diagnosis and recovery. Though rare, neuropathic pain can exacerbate behavioral and emotional disturbances, leading to additional distress. This pain likely results from disruptions in nerve pathways following surgery, necessitating specific pharmacologic interventions such as anticonvulsants. These medications are not typically included in standard PFS protocols, underscoring the need for a more individualized approach in complex cases.

This case report describes a 10-year-old boy who developed PFS following an incomplete resection of a medulloblastoma. His symptoms included mutism, irritability, sleep disturbances, emotional lability, and localized neuropathic pain at the C5 level, complicating his post-surgical recovery. Written informed consent for publication was obtained from the patient’s family.

Understanding the multifaceted nature of PFS, including associated complications such as neuropathic pain, is essential for advancing care in pediatric neuro-oncology. There is a need for ongoing research into the mechanisms and neurobiological pathways that contribute to PFS, as further insights could refine treatment protocols and enable tailored therapeutic approaches. Identifying specific risk factors might also facilitate the development of preventive surgical strategies, optimized recovery plans, and interventions to mitigate long-term disability. This case underscores the importance of early, multidisciplinary intervention to manage the neurocognitive, emotional, and physical challenges posed by PFS, ultimately aiming to improve the quality of life and long-term outcomes for affected children.

## 2. Case Report

**Figure 1:**
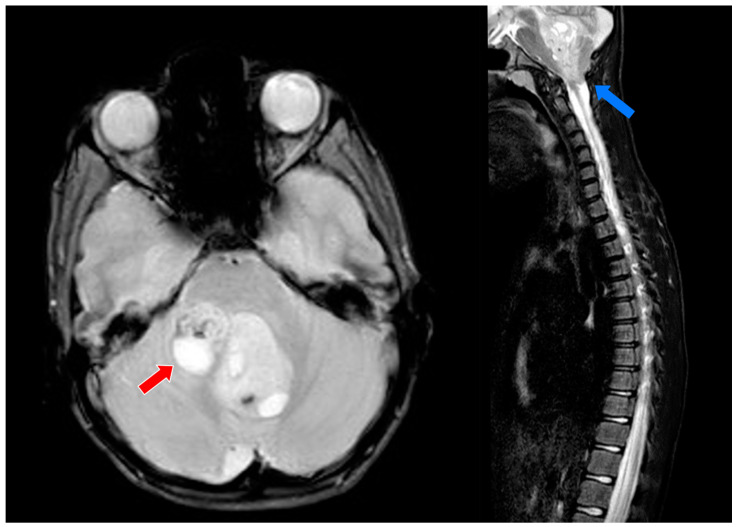
The MRI findings at the time of diagnosis of medulloblastoma in the young patient. The exam revealed a large, lobulated lesion in the right posterior cerebral fossa, appearing hypo-intense on T1-weighted imaging and hyper-intense on T2-weighted imaging, with heterogeneous gadolinium enhancement (red arrow). There was spinal cord compression at the C1 level (blue arrow), obliteration of the fourth ventricle, and cerebellar tonsillar herniation into the foramen magnum. Additionally, diffuse leptomeningeal dissemination was observed, extending ventrally and rostrally from C5 to T2 and T5 to T9 levels.

A Caucasian 10-year-old boy presented to the ED of Policlinico Hospital of Bari, Italy, for vomiting and headache that lasted for several days. A brain and spine MRI revealed a big lesion in the posterior cerebral fossa, in the right median–paramedian area, with poly-lobed margins, hypo-intense on T1-weighted imaging, and hyper-intense on T2-weighted imaging, with heterogeneous gadolinium enhancement. Spinal cord compression at C1 level was evident, and the fourth ventricle was no longer visible, with descent of cerebral tonsils into the foramen magnum. Diffuse ventral and rostral leptomeningeal dissemination was present, from C5 to T2 and from T5 to T9 levels (see Figure 1). An external derivation was inserted and subtotal surgical resection was planned a few days later. Post-operative MRI showed a residual portion of the tumor at the cerebellar medal right peduncle, with slight compression of the superior peduncle. A histological exam confirmed the suspicion of classic medulloblastoma nonSHH/nonWNT. The young boy developed mutism, sleep disturbances, emotional lability, and aggressiveness two days after surgery. Gait was compromised, but possible with double support and a wide base. The young patient complained of severe pain (NRS 10/10) involving the right arm, from the neck to the C5-C6 dermatomes. According to clinical and histopathological diagnosis, the medulloblastoma was classified as WHO grade 4. At physical examination, stiffness and pain in the right shoulder were present, with several crises of severe pain, that the patient described as a pinprick-like burning sensation at the C5-C6 dermatomes, lasting from several minutes to hours. Superficial tactile allodynia was present. A slight lack of strength in the right arm was documented at neurological examination. His weight was 30 kg.

A combined therapy with diazepam 4 mg/day split in four doses, slow-release melatonin, acetaminophen, ibuprofen, and tramadol at maximum dosage per body weight was introduced (60 mg/kg/day for acetaminophen, 40 mg/kg/day for ibuprofen, and 8 mg/kg/day for tramadol, respectively). A rehabilitation program and speech training were instituted. Mutism and gait disorders improved in a few days, but pain, irritability, and sleep disturbances were unchanged. Therefore, after a multidisciplinary discussion involving a pain therapist, a psycho-oncologist, a physiotherapist, and an oncology physician, acetaminophen, ibuprofen, and tramadol were discontinued. Diazepam was increased up to 6 mg split in four doses, combined with gabapentin (a starting dose 100 mg/day, with a daily increase of 100 mg until reaching a total dose of 300 mg/day), since a diagnosis of neuropathic pain was presumed as probable. The presence of a relevant neurological deficit, a related distribution of pain, specific descriptors (e.g., pinprick-like burning sensation), and MRI findings suggest probable neuropathic pain. Baclofen was implemented at 0.3 mg/kg, split into two doses for muscle stiffness. This combined therapy allowed for a good pain control in the following days, with reduction in the frequency of painful episodes, sleep improvement, and mood advancement. Moreover, speech training, physiotherapy, and phycosocial support for both the child and his relatives were implemented.

The patient was discharged home one week later, with the suggestion of progressive tapering of medications if symptoms were still controlled, as well as the continuation of supportive therapies. Baclofen was reduced in four weeks, diazepam was gradually interrupted after six weeks, while gabapentin was stopped after eight weeks.

## 3. Discussion

This case highlights the complex management of posterior fossa syndrome (PFS) with associated neuropathic pain following an incomplete resection of a medulloblastoma. PFS remains a challenging diagnosis due to its wide variability in clinical presentation, with symptoms often emerging within a delayed window post surgery. Recently, the Posterior Fossa Society proposed a definition for this syndrome as “postoperative pediatric cerebellar mutism syndrome”, focusing on delayed-onset mutism or speech reduction as key symptoms, often accompanied by emotional lability, hypotonia, oropharyngeal dysfunction, cerebellar motor deficits, cerebellar cognitive affective syndrome (CCAS), and brainstem dysfunction [25]. The patient in this case presented with mutism, sleep disturbances, emotional lability, aggressiveness, and gait disturbance within two days post surgery, aligning with the PFS diagnostic criteria and consistent with the literature, which identifies typical symptom onset within the first week [18].

Neuropathic pain is a particularly challenging component in this case, as it is an uncommon but impactful symptom in children with PFS. Diagnosing neuropathic pain in children is complex; young patients often struggle to describe their symptoms, requiring clinicians to rely on observation and age-appropriate descriptors. Neuropathic pain can have a substantial impact on mood, sleep, and behavior, exacerbating the cognitive and emotional symptoms of PFS [26]. The patient’s descriptors, such as “burning” and “pinprick”, and the pain’s anatomical distribution from the neck to the C5-C6 dermatomes, indicated neuropathic characteristics. These findings highlight the importance of prompt identification and intervention for neuropathic pain, as untreated pain may worsen the psychological burden on young patients and impair recovery.

Traditional analgesics often provide limited relief in neuropathic pain, necessitating the use of specific medications targeting neuropathic pathways. In this case, the initial use of common analgesics (acetaminophen, ibuprofen, and tramadol) combined with diazepam for sleep disturbances did not provide adequate symptom control, thus requiring a more comprehensive approach. Given the multifaceted nature of PFS symptoms and neuropathic pain, a multidisciplinary team was essential [27]. Pain therapists, psycho-oncologists, physiotherapists, and oncology specialists collaborated to create an individualized treatment plan, including gabapentin and baclofen, which effectively managed neuropathic pain and muscle stiffness, respectively. Gabapentin, although initially designed as an anticonvulsant, has become a first-line treatment for neuropathic pain due to its ability to modulate calcium channels and inhibit excitatory neurotransmitters [28]. This approach is increasingly supported in pediatric palliative care settings, where gabapentin has shown efficacy in symptom management and is well tolerated with minimal side effects [29]. While pediatric guidelines for gabapentin use in neuropathic pain are limited, this case underscores its value in cases of PFS complicated by neuropathic symptoms, as the gradual titration of gabapentin enabled pain relief without excessive sedation.

Muscle stiffness, often associated with cerebellar syndromes, can further limit motor function, and in this patient, baclofen was introduced as a muscle relaxant. Baclofen’s efficacy in reducing spasticity has been well documented, with benefits extending to symptom management in conditions such as cerebral palsy [30]. Combining gabapentin with baclofen allowed for a multimodal approach that minimized the need for higher doses of a single medication, reducing the risk of side effects while achieving symptom control. Studies suggest that such multimodal approaches provide synergistic benefits, optimizing symptom relief and avoiding the adverse effects associated with monotherapy, which was particularly relevant in this young patient [27].

The pharmacologic management of PFS remains debated, with few established treatment guidelines. Bromocriptine and fluoxetine, along with midazolam, zolpidem, and arpiprazole, have been proposed as potential treatments, with a recent systematic review recommending bromocriptine as a first-line agent for akinetic symptoms and fluoxetine for apathy, mood deflection, and mutism [27,31,32] (see Table 1 for the proposed drugs for PFS, as well as the mechanisms and the beneficial and adverse effects reported in the literature.). However, the known side effects of bromocriptine, including insomnia and agitation, led to its exclusion in this case to avoid exacerbating the patient’s irritability and sleep disturbances. On the other hand, fluoxetine was not chosen, since its mechanism of action limited its effects on neuropathic pain compared to gabapentin. The multimodal approach incorporating gabapentin, diazepam, and baclofen was carefully chosen to address the specific symptoms presented by this patient, and the combination allowed for lower doses of each drug, potentially minimizing adverse effects while maximizing therapeutic efficacy. This combined approach is supported by findings in pediatric palliative care, where multimodal regimens often offer the best balance of efficacy and tolerability [33].

**Figure 2:**
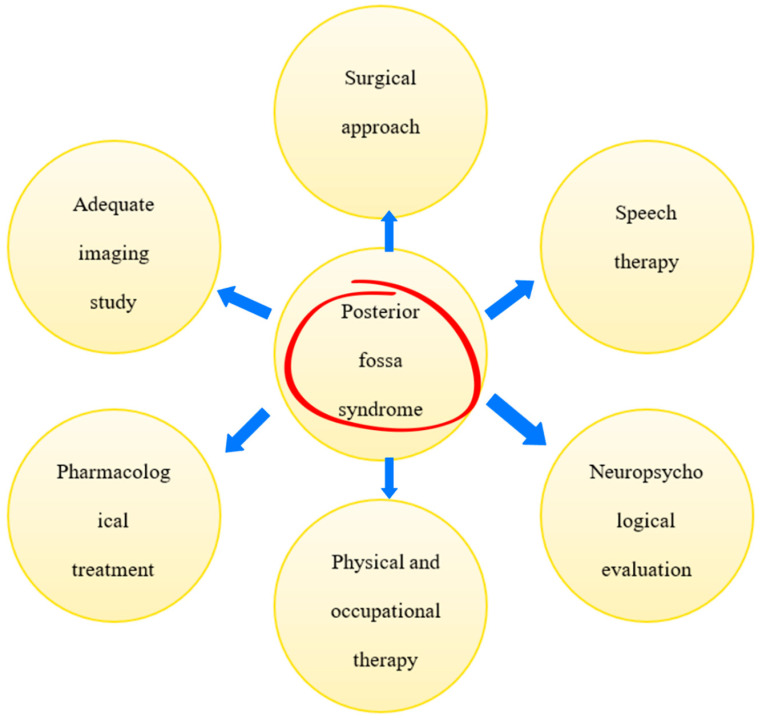
A multidisciplinary approach to posterior fossa syndrome prevention and treatment. Avoiding the aggressive intra-operative manipulation of cerebellar structures has been proposed to reduce the risk of post-operative PFS [20]. Conventional magnetic resonance imaging sequences cannot detect cerebello-cerebral diaschisis and hypertrophic olivary degeneration (which have been advocated as key pathogenetic mechanisms), and advanced MRI techniques, such as diffusion tensor imaging or brain network analysis, could be an option [21,22]. Finally, a multi-targeted approach, involving pain therapists in case of refractory pain, but also psycho-oncologists, physiotherapists, and speech therapists has been proposed as the best supportive strategy [31].

Non-pharmacologic interventions are also vital in PFS management. Physical therapy, speech therapy, and structured neuropsychological evaluations were crucial components of this patient’s care plan, contributing to progressive improvements in motor function, communication, and emotional stability. Rehabilitation is a cornerstone of PFS management, as it addresses the motor, cognitive, and affective deficits that often persist in affected children. Studies emphasize the value of augmentative and alternative communication strategies, physical and occupational therapies, and structured psychological support in helping patients and families navigate the complex recovery process [34]. Early and intensive rehabilitation, integrated with pharmacologic management, offers the best potential for meaningful recovery in PFS patients, helping to mitigate long-term deficits and improve quality of life [34] (see Figure 2).

The long-term impact of PFS on cognitive and psychosocial functioning is well-documented, as untreated or inadequately managed symptoms can significantly impair a child’s development. In a recent study, children with PFS were found to have an increased likelihood of developing obsessive–compulsive behaviors, social withdrawal, and internalizing issues one to two years after treatment compared to their peers who did not develop PFS [35]. Another review highlighted that pediatric brain tumor survivors with PFS often show lower IQ scores and social difficulties, including reduced peer relationships and limited engagement in social activities [36]. These findings stress the importance of early intervention, as these children may require long-term neuropsychological and educational support to manage the cognitive and social effects of PFS. Limiting the extent of surgery has been suggested as a strategy to minimize PFS risk; however, this approach raises concerns about potentially compromising tumor resection efficacy and long-term survival rates [37].

Neuropathic pain in PFS adds another layer of complexity to management. Painful neuropathic symptoms may lead to further mood disruptions, anxiety, and sleep disturbances, potentially amplifying the burden of PFS [26]. Given that young patients may lack the language skills to describe sensory alterations accurately, clinicians must use specialized tools and descriptors to assess pediatric neuropathic pain [26]. This case involved a careful assessment of pain descriptors such as “burning” and “pinprick”, which helped clarify the pain’s neuropathic nature [38]. Accurate pain assessment is essential, as unmanaged neuropathic pain can negatively impact rehabilitation outcomes and overall quality of life [26]. However, managing neuropathic pain in children remains challenging, as most pharmacologic recommendations for this population are extrapolated from adult data, highlighting the need for pediatric-specific research and protocols [39].

This case illustrates the importance of a multimodal approach to PFS with neuropathic pain, utilizing a combination of anticonvulsants, benzodiazepines, muscle relaxants, and comprehensive rehabilitative care. Addressing the full spectrum of symptoms—from mutism and emotional lability to neuropathic pain and motor deficits—requires a team approach involving oncology, pain management, psychology, physical therapy, and family support. By coordinating these disciplines, we were able to provide a tailored, effective treatment plan that minimized adverse effects and facilitated gradual improvements in pain, motor skills, and emotional regulation. Multimodal and multidisciplinary strategies are particularly effective in pediatric neuro-oncology, where tailored treatments are crucial to manage complex conditions such as PFS [31].

The need for ongoing research in PFS is clear. Identifying reliable, pediatric-specific treatment protocols for neuropathic pain and other PFS-related symptoms is essential. Further research could explore novel therapeutics, investigate long-term neurodevelopmental outcomes, and establish comprehensive guidelines for multidisciplinary management of PFS. Additionally, given that neuropathic pain in pediatric neuro-oncology remains underexplored, studies focused on pain mechanisms and effective pediatric pain management options could enhance outcomes for patients like the one presented in this case. The effective management of PFS, particularly in cases with associated neuropathic pain, will likely involve a combination of pharmacologic and non-pharmacologic interventions and close monitoring to optimize recovery.

To our knowledge, this case represents one of the first documented instances of PFS with a significant neuropathic pain component requiring an integrated approach involving anticonvulsants, muscle relaxants, and benzodiazepines. The outcome highlights the potential benefits of combining lower doses of multiple agents to manage symptoms more effectively without excessive side effects. This approach demonstrates that with careful assessment, individualized pharmacotherapy, and comprehensive rehabilitative support, children with complex PFS presentations can achieve improved outcomes. Continued investigation into effective PFS management strategies will be essential in providing optimal care and improving the quality of life for pediatric patients in neuro-oncology.

## Figures and Tables

**Table 1:** Dugs proposed for posterior fossa syndrome treatment, mechanisms of action, doses, indications, and adverse effects.

Drug	Dose	Mechanism of Action	Indication	Adverse Effects
Bromocriptine	2.5 mg/12 h, until maximum 15 mg/8 h	D2 receptor antagonist	Akinetic symptoms	Insomnia, agitation
Fluoxetine	10 mg/day until 20 mg/day	Selective re-uptake inhibition of serotonin	Apathy, mood deflection, and mutism	Seizures
Midazolam	0.1 mg/kg	Short-acting benzodiazepine	Dysarthria, movement disorder, sleep disorder	Eccessive sedation, respiratory depression
Zolpidem	2.5 mg/day	Non-benzopiazepine receptor modulator	Mutism and emotional lability	Insomnia
Aripiprazole	4 mg/day, then 7 mg/day	Partial dopamine agonist	Agitation, emotional lability	Not reported
Risperidone	0.5 mg/day	D2 receptor antagonist	Agitation, irritability, inconsolable crying	Not reported

## Data Availability

Data about this case report are available on request to the corresponding author due to privacy.
